# Microbial influencers and cotton leaf curl disease (CLCuD) susceptibility: a network perspective

**DOI:** 10.3389/fmicb.2024.1381883

**Published:** 2024-06-17

**Authors:** Rhea Aqueel, Ayesha Badar, Umer Zeeshan Ijaz, Kauser Abdulla Malik

**Affiliations:** ^1^Kauser Abdulla Malik School of Life Sciences, Forman Christian College (A Chartered University), Lahore, Pakistan; ^2^Water and Environment Research Group, Mazumdar-Shaw Advanced Research Centre, University of Glasgow, Glasgow, United Kingdom; ^3^National University of Ireland, University Road, Galway, Ireland; ^4^Department of Molecular and Clinical Cancer Medicine, University of Liverpool, Liverpool, United Kingdom; ^5^Pakistan Academy of Sciences, Islamabad, Pakistan

**Keywords:** co-occurrence networks, cotton leaf curl disease, plant immunity, influential nodes, keystone species, microbial ecology

## Abstract

Biotic stresses, such as plant viruses, e.g., cotton leaf curl virus (CLCuV), can alter root-associated and leaf-associated microbial diversities in plants. There are complex ecological dynamics at play, with each microbe contributing to a multitude of biotic and abiotic interactions, thus deciding the stability of the plant’s ecosystem in response to the disease. Deciphering these networks of interactions is a challenging task. The inferential research in microbiome is also at a nascent stage, often constrained by the underlying analytical assumptions and the limitations with respect to the depth of sequencing. There is also no real consensus on network-wide statistics to identify the influential microbial players in a network. Guided by the latest developments in network science, including recently published metrics such as Integrated View of Influence (IVI) and some other centrality measures, this study provides an exposé of the most influential nodes in the rhizospheric and phyllospheric microbial networks of the cotton leaf curl disease (CLCuD) susceptible, partially tolerant, and resistant cotton varieties. It is evident from our results that the CLCuD-resistant *Gossypium arboreum* possesses an equal share of keystone species, which helps it to withstand ecological pressures. In the resistant variety, the phyllosphere harbors the most influential nodes, whereas in the susceptible variety, they are present in the rhizosphere. Based on hubness score, spreading score, and IVI, the top 10 occurring keystone species in the FDH-228 (resistant) variety include *Actinokineospora, Cohnella, Thermobacillus, Clostridium, Desulfofarcimen,* and *MDD-D21. Elusimicrobia, Clostridium-sensu-stricto_12, Candidatus woesebacteria,* and *Dyella* were identified as the most influential nodes in the PFV-1 (partially tolerant) variety. In the PFV-2 (susceptible) variety, the keystone species were identified as *Georginia, Nesterenkonia, Elusimicrobia MVP-88, Acetivibrio, Tepedisphaerales, Chelatococcus, Nitrosospira,* and *RCP2-54*. This concept deciphers the diseased and healthy plant’s response to viral disease, which may be microbially mediated.

## Introduction

Microorganisms occur in the environment in either beneficial, hazardous, or neutral relationships. The nature of the microbe–microbe relationship or the host–microbe relationship is dependent on the ability of the microbe to withstand the external pressures exerted by the host or the environment it resides. As these microbes co-exist within a community, it is extremely difficult to decipher the complex ecological interactions that exist between them (Cruz et al., 2022; Geller and Levy, 2023). The plant ecosystem is one such example where microorganisms co-exist and contribute to plant health and productivity. Niche specificity and core abundance are major factors that determine the stability of a microbe in the plant ecosystem (Qiao et al., 2024). As microbes do not exist in isolation, their co-existence is highly dependent on the niche type (Barber et al., 2022). The law of competitive exclusion formulated by Gause states that two microbial species with the same niche exclude each other (Faust and Raes, 2012).

The cotton crop is devastated by the lethal cotton leaf curl virus (CLCuV), which is transmitted by the whitefly. Insect pests account for 37% of cotton yield losses, whereas the whitefly *Bemisia tabaci* is responsible for 50% of the total loss in cotton production (Oerke, 2006; Razaq et al., 2013). The genus *Gossypium* comprises 52 species, while only 4 are cultivated around the world, including *Gossypium hirsutum, Gossypium barbadense, Gossypium arboreum,* and *Gossypium herbaceum* (Ashraf et al., 2018; Hussain et al., 2020). *Gossypium hirsutum* accounts for 90% of worldwide cotton production, but it is susceptible to cotton leaf curl disease (CLCuD) (Hu et al., 2019). *Gossypium arboreum* is completely tolerant to CLCuD, but it is cultivated in less than 1% of cotton-growing areas worldwide due to its short fiber length (Edde, 2021). Conventional breeding strategies and transgenic approaches have not been able to mitigate the effects of this deadly virus. The microbiome approach has been proven successful in targeting fungal and bacterial pathogens. Biotrophic pathogens, such as the one causing CLCuD, are known to increase salicylic acid (SA) levels in infected plants as this phytohormone is found to be essential for gene-for-gene resistance, systemic acquired resistance (SAR), and reduction of disease development (Nawrath et al., 2006). Beneficial microbes from the phyllosphere can also switch on plant defense responses. Thus, plant immunity-boosting non-pathogenic microbiota is the new tool for conferring disease resistance in host plants (Legein et al., 2020).

Integrative metagenomics provides insights into the microbial community networks and ecological processes involved in biogeochemical cycles. It is still unknown how, under viral pathogen attack, the complex microbial communities interact with one another in the plant microbiome. The fields of genomics and ecology are brought together by network inference strategies, which aid in deciphering the relationships between more than two nodes involved in a particular network based on abundance data (Veiga et al., 2010). Furthermore, network topologies are useful for identifying the keystone species, i.e., those that play a pivotal role and, if perturbed, lead to maximum disruption in that network. In general, the keystone species were identified using a network property called the hubness score (Layeghifard et al., 2019), which seemed to correlate better with the properties of the ecosystem under study than looking at either the abundant or the prevalent species. A recent advancement in terms of incorporating local, semi-local, and global centrality measures, under the framework of integrated value of influence (IVI) (Salavaty et al., 2020) that also implicitly incorporates the hubness score, is shown to produce promising properties of the network while reducing the analytical biases. Therefore, we have incorporated the network statistics to explore how disease susceptibility correlates with some of these properties. We have employed the 16S rRNA gene amplification to unravel the spatial distribution patterns and complexities of the cotton microbiome in the CLCuD susceptible, partially tolerant, and resistant cotton varieties infected with CLCuV.

## Materials and methods

### Sample collection

**Figure 1:**
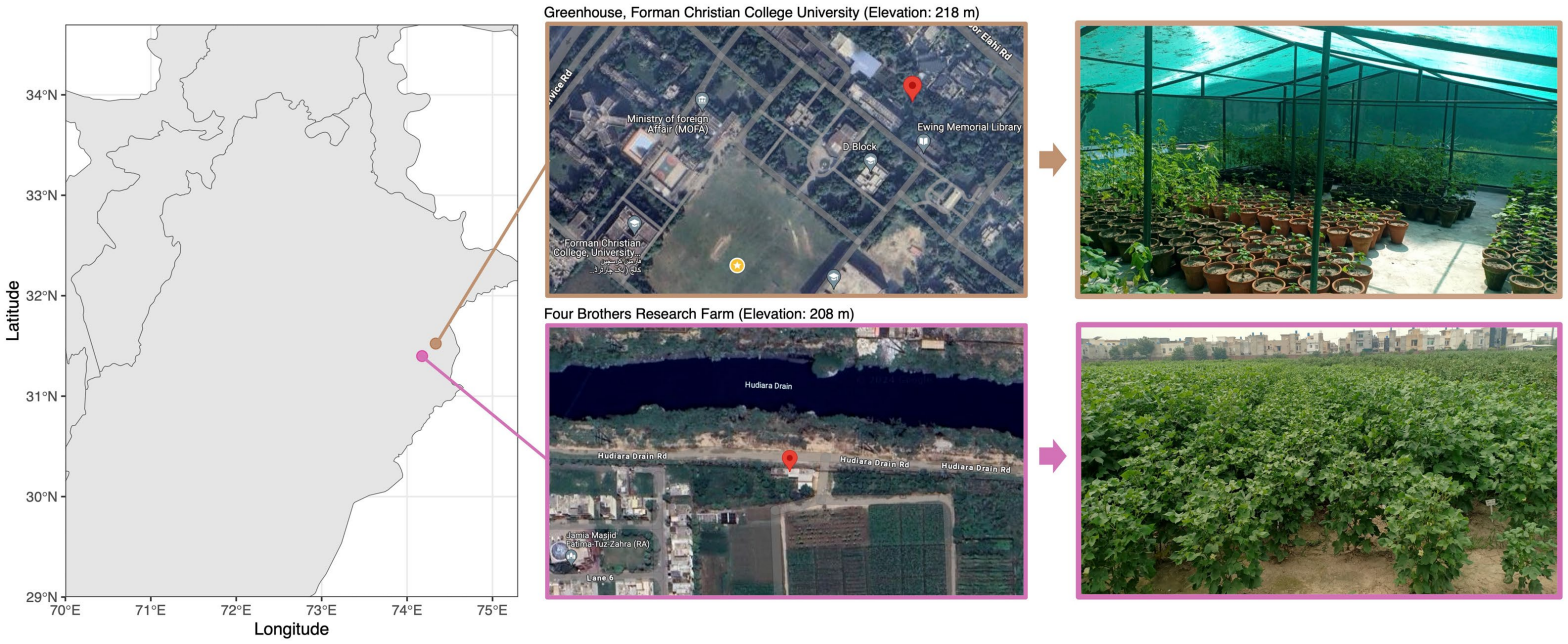
Cotton plant sampling sites along with satellite and location imagery.

Three cotton varieties were selected for the study: PFV-2 (CLCuD-susceptible *Gossypium hirsutum*), PFV-1 (CLCuD partially tolerant *Gossypium hirsutum*), and FDH-228 (CLCuD-resistant *Gossypium arboreum*). The *Gossypium hirsutum* plants (5 each variety: PFV-2 and PFV-1) were sampled from Four Brothers Research Farm (31.399043° N, 74.175621° E) and the *Gossypium arboreum* (FDH-228) plants were sampled from the greenhouse at Forman Christian College (A Chartered University) (31.523565 N, 74.335380 E). Both sites (Figure 1) are located in Lahore, which has a semi-arid climate with an annual average rainfall of 628.8 mm. Leaf samples were collected in autoclave bags and were stored in ice until they were brought to the lab. Roots with adhered rhizospheric soil were stored in 50 mL falcon tubes and stored in ice until they were brought to the lab. The samples were stored at −80°C until further processing.

### DNA extraction from soil, root, and leaf compartments

The study was aimed at the analysis of four plant compartments, namely the leaf endophytic, leaf epiphytic, root endophytic, and rhizospheric region. For DNA extraction from the leaf epiphytic region, the leaves were washed with 1X T.E. buffer containing 0.2% Triton X. The wash was filtered through a 0.2 μM sterile filter paper, and the filter paper was used for DNA extraction. The leaf was washed with 70% ethanol followed by 3% bleach, and multiple washings were given with sterile distilled water (SDW) to get rid of the leaf epiphytes. The leaf sample (100 mg) was crushed in PBS buffer using a pestle and mortar. The resultant solution was collected in a falcon tube and used for DNA extraction. Rhizospheric soil (up to 3 mm around the root surface area) was separated by sonication of roots in PBS buffer. The roots were separated and sterilized by washing with 70% ethanol and 3% bleach once and SDW several times to eliminate rhizospheric bacteria. The root (100 mg) was macerated in PBS buffer using a pestle and mortar and was collected in a falcon tube. Total DNA was extracted using the FastDNA Spin Kit for Soil (MP Biomedicals, California, USA) according to the manufacturer’s instructions. Samples were homogenized in the FastPrep instrument for 40 s at a speed setting of 6.0. The DNA was eluted in 30 μL of elution buffer.

### PCR amplification and high-throughput sequencing

A total of 60 DNA samples (5 replicates of 3 varieties x 4 plant compartments) were amplified using the primer pair 341F (5′-TCGTCGGCAGCGTCAGATGTGTATAAGAGACAGCCTACGGGNGGCWGCAG-3′) and 805R (5′-GTCTCGTGGGCTCGGAGATGTGTATAAGAGACAGGACTACHVGGGTATCTAATCC-3′) (Herlemann et al., 2011). The primer pair contained Illumina adapter overhang sequences for the amplification of 16S rRNA hypervariable region V3-V4. The PCR reaction mixture contained 12.5 μL of KAPA HiFi HotStart ReadyMix (Roche), 1 μL from 10 μM of each primer, 1 μL of each mPNA and pPNA blocker, 2 μL of metagenomic DNA template (10 ng/μl), and remaining volume was made up to 25 μL with nuclease-free water. The PCR conditions were set as follows: 95°C for 5 min (initial denaturation), followed by 35 cycles of 94°C for 1 min (denaturation), 55°C for 1 min (annealing), 72°C for 1 min, and 30 s (extension) with a final extension of 10 min at 72°C. PCR reactions were cleaned up with AMPure® XP beads. The samples were sent to Macrogen, Inc. Seoul, South Korea, for amplicon sequencing on an Illumina MiSeq platform.

### Network inference

The 16S rRNA sequences were processed with the QIIME2 pipeline with the dataset given by Aqueel et al. (2023) and revisited in this study. In brief, the Deblur algorithm (Amir et al., 2017) within the QIIME2 platform (version 2019.7.0) was used to recover 38,120 amplicon sequence variants (ASVs). The sequencing reads were imported to QIIME2 format and were quality trimmed with a minimum Phred quality score of 20. This was followed by using the qiime deblur denoise-other plugin with parameters --p-trim-length 280 --p-min-size 2 --p-min-reads 2 to generate ASVs. As a preprocessing step, the Deblur method also filters out any sequences that are not found in the reference SILVA SSU Ref NR database v138 (Quast et al., 2012), which is additionally used in qiime feature-classifier plugin to assign taxonomy to each ASV. This yielded a *n* = 59 (sample) X 38,120 (ASV) abundance table with summary statistics of sample-wise reads matching to ASVs as follows: [1st Quartile:7,979; Median:15,522; Mean: 14;565; 3rd Quartile:21,387; and Maximum: 27,839]. The detailed statistics from different bioinformatics steps are given in Supplementary Table S6. Using the SILVA taxonomy, the ASVs were collated at the genus level (849 genera), with three tables extracted for each of the varieties, FDH-228 (*n* = 17), PFV-1 (*n* = 17), and PFV-2 (*n* = 16), respectively. To find the relationship between the genera, rather than using the traditional correlation analyses, we have used a recent approach by Lovell et al. (2015), which showed that variables that have nearly constant ratios in all samples are highly correlated. Therefore, Phi statistics is calculated where the $var\left(\frac{clr\left(x\right)}{clr\left(y\right)}\right)$ for two taxa x and y is essentially constant. clr(x) is the centralized log-ratio transform of the abundance table. Before using the phi statistics, we preprocessed the abundance tables for each variety using the standard protocols given at^1^, where the R package zComposition (Palarea-Albaladejo and Martín-Fernández, 2015) is applied (cmultRepl() function with the argument method = “CZM”) to replace 0 s in the abundance table with an estimate of the probability that the zero is not 0. Afterward, from the CoDaSeq package, codaSeq.clr() function is applied to calculate the centralized log transform, and then propr.phisym() function is used to calculate the phi statistics and retain those taxa pairs where the phi statistics is <0.1 as recommended by the authors. For comparing network-wide statistics, we have employed the standard ANOVA using aov() function available in R.

### Network-wide statistics

Having obtained the network topology for all three varieties, we have calculated several network-wide statistics using numerous R packages, including igraph (Csardi and Nepusz, 2006), influential (Salavaty et al., 2020), and centiserve (Jalili et al., 2015). We have used the statistics with the definitions given in the Supplementary materials.

## Results

### The most influential co-occurring species among CLCuD susceptible, partially tolerant, and resistant varieties

**Figure 2:**
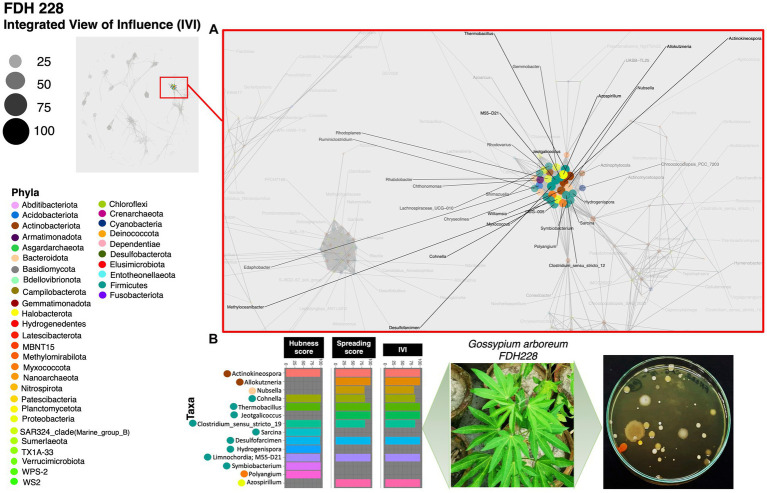
Network inferred for *Gossypium arboreum* FDH-228 samples using the phi statistics. **(A)** The complete networks highlighting the regions with the most influential nodes colored by their taxonomic assignment at the phylum level; **(B)** The top 10 important nodes along with their scores based on hubness Score, Spreading Score, and their composite measure IVI. The plate shows the bacterial diversity of microbes extracted from the plant.

**Figure 3:**
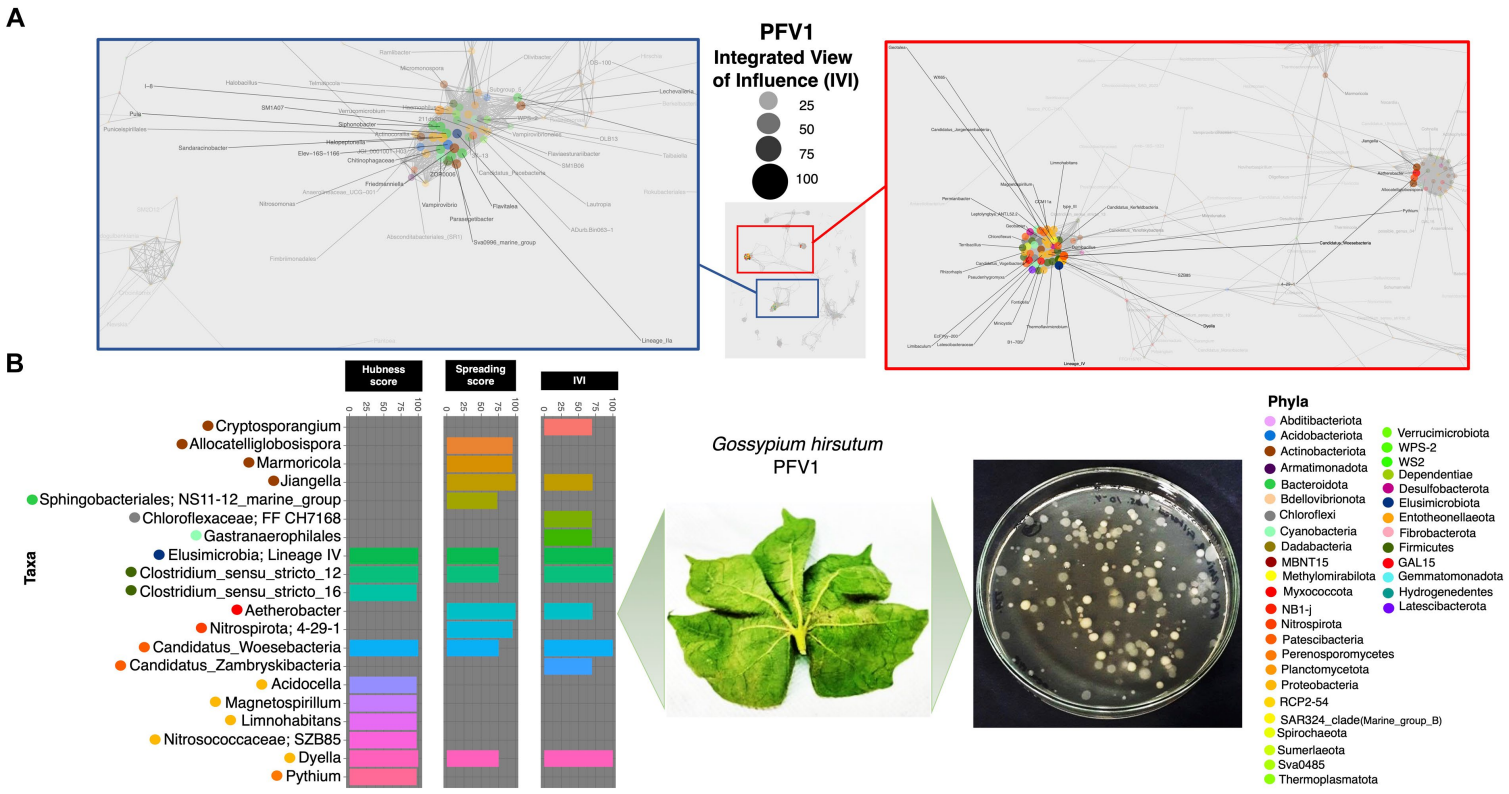
Network inferred for *Gossypium hirsutum* PFV-1 samples using the phi statistics. **(A)** The complete networks highlighting the regions with the most influential nodes colored by their taxonomic assignment at the phylum level. **(B)** The top 10 important nodes along with their scores based on hubness Score, Spreading Score, and their composite measure IVI. The plate shows the bacterial diversity of microbes extracted from the plant.

**Figure 4:**
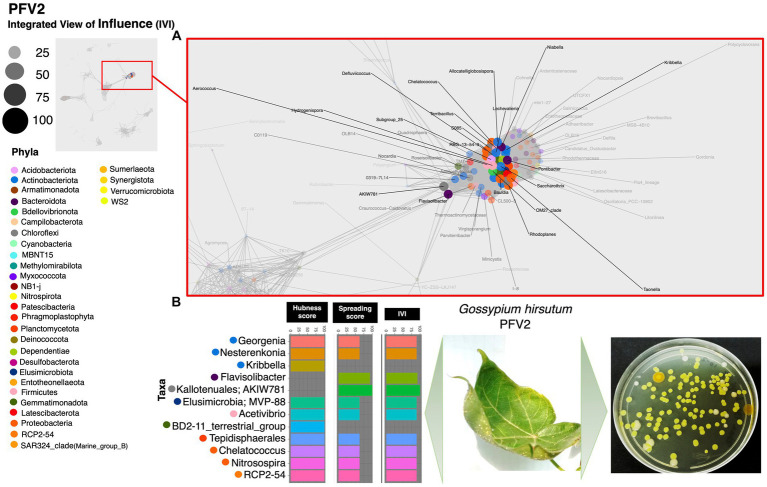
Network inferred for *Gossypium hirsutum* PFV-2 samples using the phi statistics. **(A)** The complete networks highlighting the regions with the most influential nodes colored by their taxonomic assignment at the phylum level. **(B)** The top 10 important nodes along with their scores based on hubness Score, Spreading Score, and their composite measure IVI. The plate shows the bacterial diversity of microbes extracted from the plant.

Based on the hubness score, we have listed the top 10 keystone species in the network of the three cotton varieties. Supplementary Table S2 depicts the top 10 co-occurring bacterial species in color. The resistant variety (FDH-228) has fewer interacting phyla with very strong interactivity and hegemony, while the partially tolerant and susceptible varieties (PFV-1 and PFV-2) had preponderances of different unique phyla, more than the resistant variety. Phylum *Actinobacteriota, Firmicutes*, and *Proteobacteria* were observed in the network of FDH-228, where the abundantly occurring phylum was *Firmicutes* in the top 10 keystone species (Figure 2). In PFV-1, phyla including *Patescibacteria, Elusimicrobiota, Firmicutes*, and *Proteobacteria* were observed (Figure 3). Interestingly, there was an abundance of many unique phyla in the susceptible variety PFV-2, which included *Actinobacteriota, Bacteroidota, Bdellovibrionota, Dependentiae, Gemmatimonadota, Latescibacterota, Proteobacteria, RCP2-54, SAR 324 clade (Marine_group_B), Firmicutes, Elusimicrobiota, Chloroflexi*, and *Acidobacteriota* (Figure 4). It is important to note that only the network of FDH-228 shows *Firmicutes* in abundance, whereas the top 10 keystone bacterial genera of *Gossypium hirsutum* varieties have very little abundance of this phylum. According to the three selected network statistics (hubness score, spreading score, and IVI) based on which the top 10 occurring keystone species were identified, *Actinokineospora, Cohnella, Thermobacillus, Clostridium, Desulfofarcimen,* and *MDD-D21* were observed to be the most influential nodes of the network of the CLCuD-resistant variety FDH-228. The keystone species for the partially tolerant variety were identified as *Elusimicrobia, Clostridium-sensu-stricto_12, Candidatus woesebacteria,* and *Dyella*. Finally, *Georginia, Nesterenkonia, Elusimicrobia MVP-88, Acetivibrio, Tepedisphaerales, Chelatococcus, Nitrosospira,* and *RCP2-54* were characterized as the most influential nodes in the network of the susceptible variety.

### Compartment-wise differences of influential nodes within a particular variety

**Figure 5:**
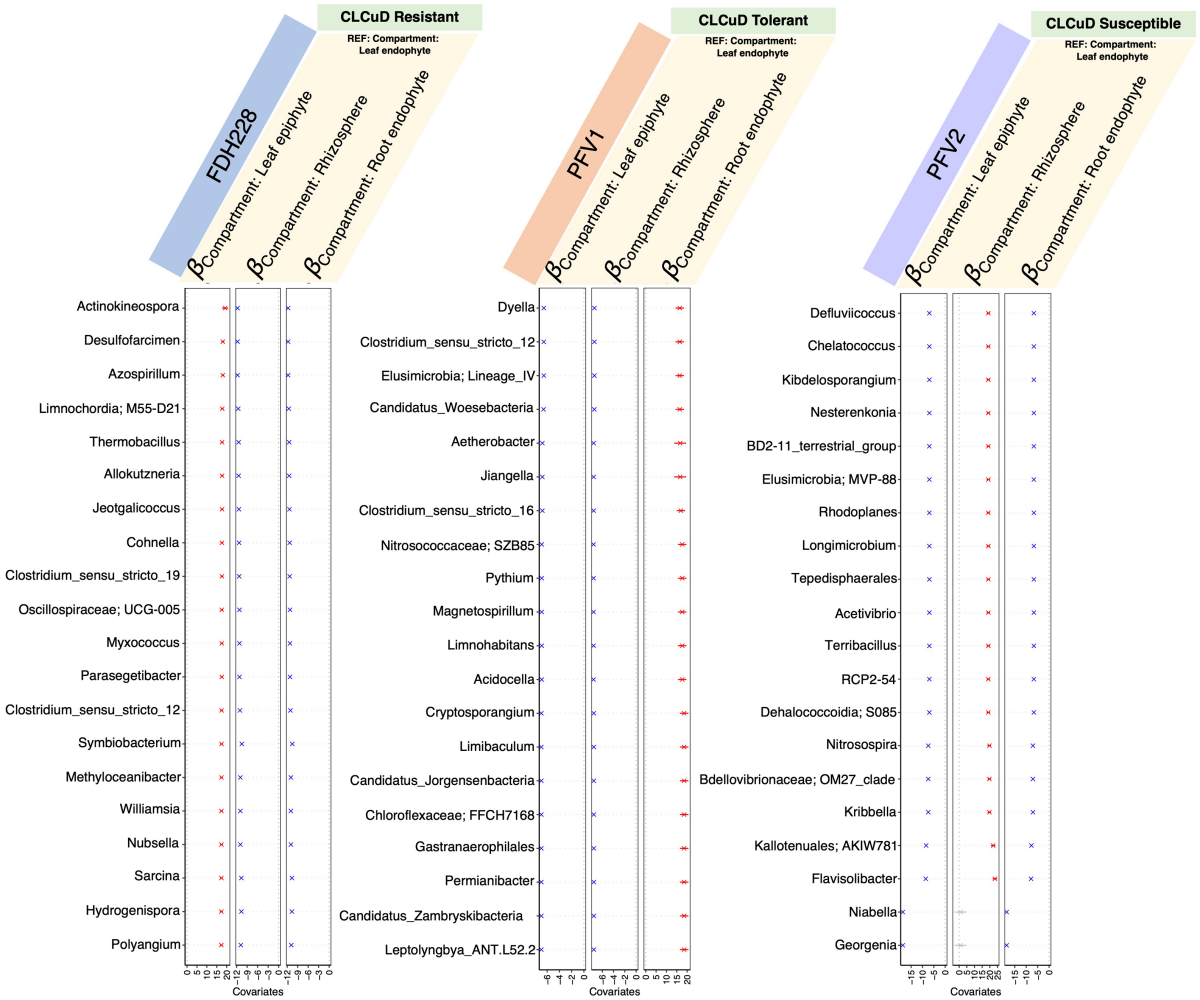
𝜷− coefficients returned from the GLLVM procedure for covariates considered in this study and the top 20 most influential nodes returned for different varieties (from left to right, these are FDH-228, PFV-1, and PFV-2, respectively). Those coefficients that are positively associated with the microbial abundance of particular genera are represented in red color while those that are negatively associated are represented in blue color, respectively. Non-significant associations, if any, are represented in gray color. For categorical variables, one level acts as a reference and is annotated with “REF.”

To explore the positive and negative associations of the microbiota with the selected phyllospheric and rhizospheric plant compartments, we used the *generalized linear latent variable model* (GLLVM) model approach (Figure 5). As compared to the leaf endophyte, the top 10 keystone species were positively correlated with the leaf epiphytic compartment in FDH-228, whereas they were negatively correlated and followed the same decreasing trend in the *Gossypium hirsutum* varieties, PFV-1 and PFV-2. Compared to the leaf endophyte, the keystone species in the rhizosphere have a similar pattern in the resistant (FDH-228) and partially tolerant (PFV-1) varieties, as they show a negative correlation. The keystone species in PFV-1 were positively associated with the root endophytic compartment.

### Network-wide statistics associated with CLCuD susceptibility

**Figure 6:**
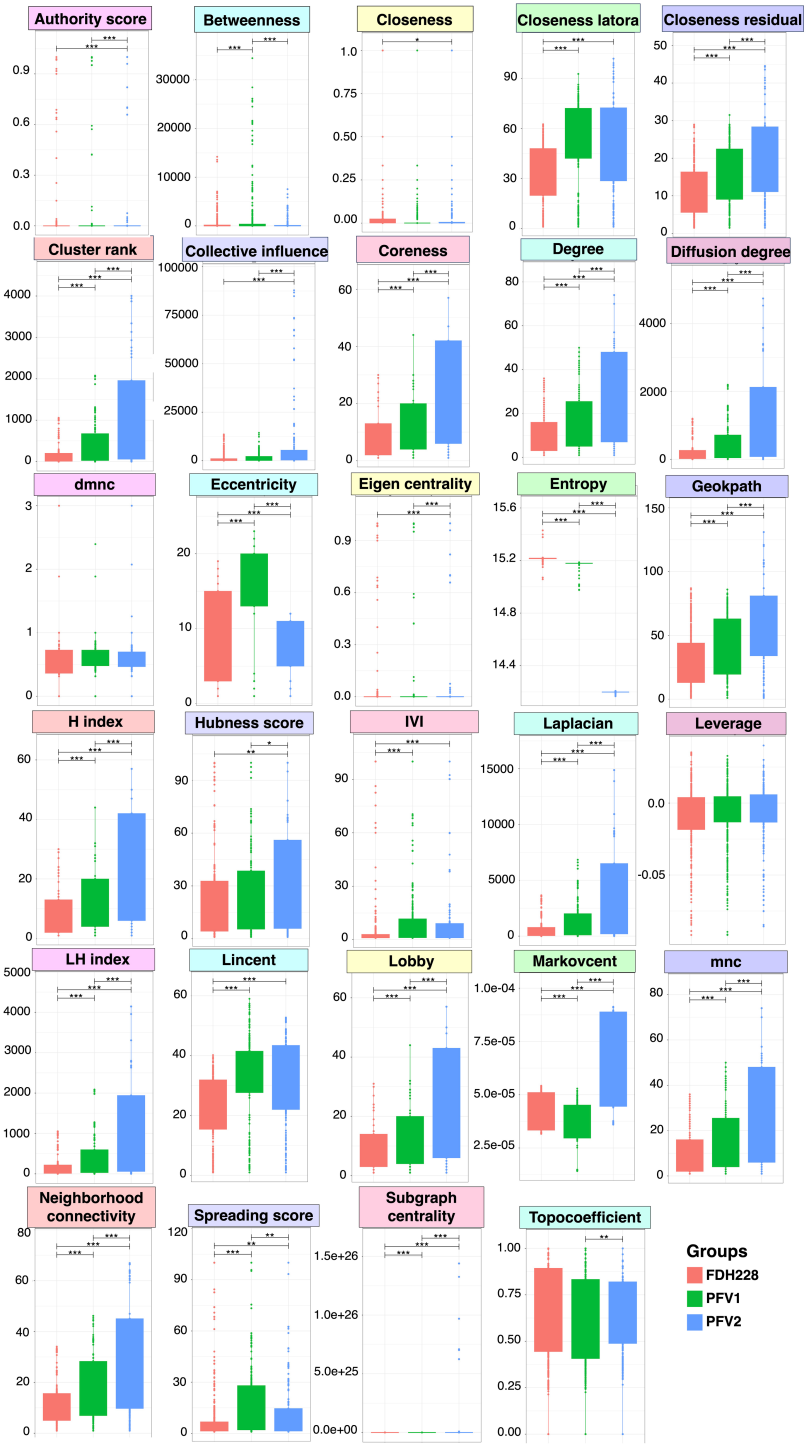
A comparison of network-wide statistics for the networks obtained for FDH-228, PFV-1, and PFV-2 varieties. Lines for panels A and B connect two sample groups at statistically significant levels (according to ANOVA) indicated by asterisks as * (*p* < 0.05), **(*p* < 0.01), or ***(*p* < 0.001). The raw statistics are available as Supplementary Table S3 (FDH-228), Supplementary Table S4 (PFV-1), and Supplementary Table S5 (PFV-2), respectively.

Network statistics are the fundamental way to understand the underlying nature of the most influential nodes in the network, and we have revealed the positive/negative trends associated with CLCuD susceptibility for 29 different network statistics (Figure 6) with their definitions given in the Supplementary materials. The most influential nodes in the three networks for FDH-228 (resistant), PFV-1 (partially tolerant), and PFV-2 (susceptible) were identified using the Integrative Value of Influence (IVI) measure, and the statistics revealed that PFV-1 has the highest IVI value where two networks were identified to have the most influential nodes (Figure 3). Hubness score is indicative of how powerful those nodes are in their ecosystem. The hubness score and H-index also depicted an increase in trend from FDH-228 to PFV-2. Laplacian, Local H (LH) index, Mean Neighborhood Connectivity (MNC), and the Lobby index showed the highest value for susceptible variety PFV-2. The spreading potential of a node in each network was explained by the spreading score, where the PFV-1 network had the highest spreading score, followed by the networks for PFV-2 and FDH-228. The closeness residual statistic specifies how close the influential nodes are within a network. The influential nodes were closest in the network of the susceptible variety PFV-2, with the order decreasing from PFV-1 to FDH-228. A connection between the local and semi-local characteristics of a node is indicated via the cluster rank statistic, and it shows an increasing trend from resistant to susceptible variety. Collective influence is focused on highlighting the minimum set of influential nodes in the network, where PFV-2 only shows a minimal increase as compared to PFV-2 and PFV-1. A larger coreness value is indicative of the fact that the nodes are more centrally located in the network: networks of PFV-2 and PFV-1 have larger coreness values than those of FDH-228. The degree and diffusion degree have the same trend, with the value of networks in PFV-2 being the highest. Entropy in network statistics aims to explain the degree of disorder or complexity of the network. Higher entropy values indicate lesser information gain from the networks, as are indicated by values for the networks of FDH-228 and PFV-1. Topocoefficient indicates how many nodes are shared with the neighboring nodes, and this statistic is observed to be the highest for the FDH-228 network, followed by the networks for PFV-1 and PFV-2.

## Discussion

The term “microbiome influencer” holds prime importance in microbial ecology (Paul et al., 2022). It is the most influential node in an ecosystem that contributes to crop vigor and resilience as opposed to its most abundant species. The core community does hold primordial significance (Toju et al., 2018), but the stability of the microbial influencers is a major factor in the plant’s response to biotic or abiotic stressors (Qiao et al., 2024). Viruses are a major threat to plants worldwide, and the cotton leaf curl virus (CLCuV) is one of the deadliest viruses affecting the cotton crop (Mahmood-ur-Rahman et al., 2012). Crop phenology is majorly dependent on the microbiomes and mycobiomes that inhabit the internal and surrounding environment (Sharma et al., 2019; Ginnan, 2020). Rather than existing in isolation, microorganisms co-occur in ecological networks that determine the stability of the entire ecosystem and thus the plant’s response to biotic/abiotic stresses (Afridi et al., 2022). The drivers of these networks are the taxa termed the “most influential nodes” (Mukhtar et al., 2023).

In the FDH-228 network, more nodes have an IVI of 100, whereas in the PFV-2 network, only *Chloroflexi* has an IVI of 100. Members of the phylum *Chloroflexi* possess anaerobic fermentation potential and have been discovered in diverse habitats such as hot springs, sediments, and anaerobic sludge digesters (Hug et al., 2013; Xia et al., 2016; Petriglieri et al., 2018; Bovio et al., 2019). If the influential node stands prominent against all other nodes, i.e., if the difference is huge, then any perturbation in that influential node should propagate and disrupt the network far more, which is evident for PFV-2. On the contrary, in the FDH-228 resistant variety, on average, there are many influential nodes, with authority predominantly shared among firmicutes. We hypothesize that the community is robust against perturbations and should manage challenges better as a result of the decentralization of authority.

From GLLVM, it is quite apparent that the influential nodes all have consistently similar signs, whether all positive or all negative, which are associated with a particular compartment as compared to the reference. This seems to suggest that these species form part of a cohort that is increasing or decreasing globally as a cohort rather than exhibiting local changes in a few members. Compartment-wise network analysis revealed that drought stress disrupted the microbial network in the root endosphere, which contained the most influential nodes, compared to the phyllosphere and rhizosphere (Gao et al., 2022).

Microbial communities inhabiting different plant compartments exhibit varying capabilities owing to selection pressures associated with the compartment type. As this research aims to identify bacteria that could serve as biocontrol agents, it was necessary to screen all plant compartments for microbes that may possess disease-suppressing abilities. The recruitment of root-associated microbes relies heavily on the root exudates secreted by the plant roots (Massalha et al., 2017) and also the environmental factors such as soil pH, salinity, soil type, soil structure, soil moisture, and soil organic matter (Sindhu et al., 2022). Conversely, leaf-associated bacteria exhibit a low species richness as the phyllosphere is relatively nutrient-poor compared to the highly fertile rhizosphere. The leaf surface constitutes an inhospitable environment that is characterized by fluctuations in temperature, moisture level, and nutrient availability (Thapa and Prasanna, 2018). Phyllospheric bacteria, therefore, possess the ability to maintain environmental homeostasis by producing secondary metabolites or exogenous polysaccharides to aid in the survival of the host plant (Jackson et al., 2015).

It is interesting to note that in the partially tolerant PFV-1 network, the most influential nodes are root and leaf endophytic bacteria. It can be observed that the microbial influencers are in abundance in the network of the highly susceptible variety’s rhizosphere. The rhizosphere is known to harbor commensals and recruits them from the surrounding environment (Bulgarelli et al., 2013; Müller et al., 2016). In a previous study, rhizospheric microbial taxa and influential nodes were enriched in plants infected with the soil-borne yellow mosaic virus. The presence of beneficial taxa, including nitrogen fixers, such as members of *Bradyrhizobiaceae, Xanthomonadaceae, Sphingomonadaceae*, and *Comamonadaceae,* in the co-occurrence networks of infected wheat plants reveals that the pathogen is interdependent on the beneficial microbes that have maintained the ecological niche in the presence of disease (Wu et al., 2021). *Azospirillum*, one of the top influential nodes in the FDH-228 co-occurrence network, is a gram-negative nitrogen fixer capable of IAA, CK, and GA_3_ production (Cassán and Diaz-Zorita, 2016). *Georgenia*, one of the influencers in the susceptible PFV-2 network, is a heterotroph capable of aerobic denitrification and has been previously isolated from deep-sea sediments and forest soils (Li et al., 2007; Wang et al., 2015; Rajta et al., 2022). *Acetivibrio* is an obligate anaerobe (Charoensuk et al., 2019) and *Nitrosospira* is an ammonia oxidizer (Koike et al., 2022), and both belong to the PFV-2 co-occurrence network.

In the resistant variety, the phyllosphere is home to the most influential nodes present in the network. The leaf epiphytic region is dominated by the top influencers, which is in accordance with our previous findings, where the SA-producing bacteria isolated from the phyllosphere of the FDH-228 variety conferred disease resistance against CLCuD in the susceptible variety (Aqueel et al., 2023). The phyllosphere microbiota are the most selected microbes and partake in nutrient cycling due to their specialized adaptations to climate change (Dorokhov et al., 2018; Cavicchioli et al., 2019; Koskella, 2020). They are also crucial for immune priming and pathogen elimination (Bell et al., 2019; Chen et al., 2020).

## Conclusion

Our co-occurrence network analyses of the CLCuD-infected cotton plants with varying levels of susceptibility have revealed that the microbiome influencers show a consistent response in different compartments. The network of the resistant *Gossypium arboreum* possesses many influential taxa from the phylum *Firmicutes*. The revelation of these networks can help us understand the crosstalk between the plant genotype and microorganisms inhabiting various plant compartments under pathogenic attack. This will aid in the utilization of these ‘influential ecological drivers’ for viral disease suppression in cotton.

## Data availability statement

The datasets presented in this study can be found in online repositories (European Nucleotide Archive under the project accession number PRJEB67645). The meta data associated with the samples can be found in the Supplementary material.

## Author contributions

RA: Formal analysis, Writing – original draft, Conceptualization, Data curation, Investigation, Visualization. AB: Formal analysis, Data curation, Investigation, Visualization, Writing – review & editing. UZI: Formal analysis, Funding acquisition, Methodology, Software, Supervision, Writing – original draft. KAM: Conceptualization, Funding acquisition, Methodology, Project administration, Supervision, Writing – review & editing.

## Footnotes

^1^https://github.com/ggloor/CoDaSeq

## Supplementary material

SUPPLEMENTARY TABLE S1 Metadata associated with the deposited sequences.

SUPPLEMENTARY TABLE S2 Co-occurrence network obtained at genera level using phi statistics for FDH-228, PFV-1, and PFV-2. Furthermore, the top 10 interacting species are highlighted for each variety.

SUPPLEMENTARY TABLE S3 Network-wide statistics for FDH-228 network.

SUPPLEMENTARY TABLE S4 Network-wide statistics for PFV-1 network.

SUPPLEMENTARY TABLE S5 Network-wide statistics for PFV-2 network.

SUPPLEMENTARY TABLE S6 Statistics of sample reads during numerous bioinformatics steps including the application of Deblur algorithm.
