# Supplementary material for: Microbial influencers and cotton leaf curl disease (CLCuD) susceptibility: a network perspective
Source: Front Microbiol. 2024 Jun 17;15:1381883. doi: 10.3389/fmicb.2024.1381883 (PMC11215052; doi:10.3389/fmicb.2024.1381883)
Supplement: SUPPLEMENTARY TABLE S1 — Metadata associated with the deposited sequences. [file Data_Sheet_1.zip › Supplementary Methods.PDF]

Network-wide statistics

Having obtained the network topology for all three varieties, we have calculated several network wide statistics using numerous R packages including igraph [1], influential [2], and centiserve [3]. We have used the statistics given in Supplementary Table 1.

Supplementary Table 1: The network-wide statistics along with their description and the R packages used.

Measure	Description	Package	Function
Degree centrality	The degree centrality of a node is simply its degree - the number of edges it has. The higher the degree, the more central the node is.	igraph	igraph::degree()
Closeness centrality	How close a given node is to any other node, and defined as the inverse of the average of the shortest path between a node and all other nodes	igraph	igraph::closeness()
Betweenness centrality	The number of shortest paths passing through a node.	igraph	igraph::betweenness()
Eigenvector centrality	The importance of a node is recursively related to the importance of the nodes pointing to it. A higher value implies that a node's neighbours are more prestigious than the neighbours of other nodes	igraph	igraph::eigen_centrality()
Subgraph centrality	It sums up all closed walks weighting them by the inverse factorial of its length	igraph	igraph::subgraph_centrality()
Coreness	The k-core of a graph is a maximal subgraph in which each vertex has at least degree k. The coreness of a vertex is k if it belongs to the k-core but not to the (k+1)-core	igraph	igraph::coreness()
Kleinberg's authority centrality score	The authority scores of vertices are defined as principal eigenvector of t(A)*A, where A is the adjacency matrix of the network	igraph	igraph::authority_score()

Eccentricity	Shortest path distance from the farthest other node in the graph	igraph	igraph::centrality()
Neighborhood Connectivity	Average number of edges connected to immediate neighbors	influential	influential::neighborhood.connectivity()
H-index	Semi-local centrality measure inspired from its application in assessing the impact of researchers	influential	influential::h_index()
Local H-index	An improved version of the H-index centrality that leverages the H-index to the second order neighbors of a node	influential	influential::lh_index()
Spreading Score	Spreading score reflects the spreading potential of each node within a network, and is calculated as $Spreading_{score_i} = (NC_i' + CR_i')(BC_i' + CI_i')$ where NC_i', CR_i', BC_i', CI_i' are range normalized neighborhood connectivity, cluster rank, betweenness centrality, and collective influence of node *i*.	influential	influential::spreading.score()
Collective Influence	A global centrality measure that calculates the product of the reduced degree (degree – 1) of a node and the total reduced degree of all nodes at a distance d from the node.	influential	influential::collective.influence()
Cluster Rank	ClusterRank is a local centrality measure that makes a connection between local and semi-local characteristics of a node and at the same time removes the negative effects of local clustering.	influential	influential::clusterRank()
Integrated View of Influence (IVI)	A method for the identification of network most influential nodes in a way that captures all network topological dimensions, and is calculated as	influential	influential::ivi()

	$IVI_i = (Hubness_{score_i})(Spreading_{score_i})$		
Hubness Score	Reflects the power of each node in its surrounding environment, and is calculated as $Hubness_{score_i} = DC'_i + LH'_{index_i}$, where DC'_i and $LH'_{index_i}$are ranged normalized degree centrality and local H index of node i, respectively.	influential	influential::hubness.score(ig)
Topological coefficient	The extent to which a node shares neighbours with other nodes.	centiserve	centiserve::topocoefficient()
Diffusion degree	The cumulative contribution score of the node itself and its neighbors.	centiserve	centiserve::diffusion.degree()
Density of maximum neighborhood component (DMNC)	It explores and identifies hubs/essential nodes in a network based on [4]	centiserve	centiserve::dmnc()
Geodesic K-path centrality	Counts neighbours as those that are on a geodesic path less than "k" away	centiserve	centiserve::geokpath()
Lin centrality	It considers closeness not as the inverse of a sum of distances, but rather than the inverse of the average distances	centiserve	centiserve::lincent()
Lobby Index	The l-index or lobby index of a node x is the largest integer k such that x has at least k neighbors with a degree of at least k	centiserve	centiserve::lobby()
Markov centrality score	The Markov centrality score uses the concept of a random walk through the graph to calculate the centrality of each vertex	centiserve	centiserve::markovcent()
Latora closeness centrality	This variant (sum of inversed distances to all other nodes instead of the inversed of the sum of distances to all other nodes) applicable to both connected and unconnected graphs	centiserve	centiserve::closeness.latora()

Entropy centrality	Measures centrality of nodes depending on their contribution to the entropy of the graph	centiserve	centiserve::entropy()
Laplacian centrality	It is defined as the drop in the Laplacian energy (i.e. sum of squares of the eigenvalues in the Laplacian matrix) of the graph when the vertex is removed.	centiserve	centiserve::laplacian()
Leverage centrality	Leverage centrality considers the degree of a node relative to its neighbors and operates under the principle that a node in a network is central if its immediate neighbors rely on that node for information.	centiserve	centiserve::leverage()
Maximum neighborhood component (MNC)	The neighborhood of a node v, nodes adjacent to v, induce a subnetwork N(v).The score of node v, MNC(v), is defined to be the size of the maximum connected component of N(v). The neighborhood N(v) is the set of nodes adjacent to v and does not contain node v.	centiserve	centiserve::mnc()
Residual closeness centrality	Calculates the closeness of a vertex using [5]	centiserve	centiserve::closeness.residual()

References:

[1] Csardi, G., & Nepusz, T. (2006). The igraph software package for complex network research. InterJournal, complex systems, 1695(5), 1-9.

[2] Salavaty, A., Ramialison, M., & Currie, P. D. (2020). Integrated value of influence: an integrative method for the identification of the most influential nodes within networks. Patterns, 1(5).

[3] Jalili, M., Salehzadeh-Yazdi, A., Asgari, Y., Arab, S. S., Yaghmaie, M., Ghavamzadeh, A., & Alimoghaddam, K. (2015). CentiServer: a comprehensive resource, web-based application and R package for centrality analysis. PloS one, 10(11), e0143111.

[4] Lin, C. Y., Chin, C. H., Wu, H. H., Chen, S. H., Ho, C. W., & Ko, M. T. (2008). Hubba: hub objects analyzer—a framework of interactome hubs identification for network biology. Nucleic acids research, 36(suppl_2), W438-W443.

[5] Dangalchev, C. (2006). Residual closeness in networks. Physica A: Statistical Mechanics and its Applications, 365(2), 556-564.
